# Evaluation of spoligotyping, SNPs and customised MIRU-VNTR combination for genotyping *Mycobacterium tuberculosis* clinical isolates in Madagascar

**DOI:** 10.1371/journal.pone.0186088

**Published:** 2017-10-20

**Authors:** Rondroarivelo Rasoahanitralisoa, Niaina Rakotosamimanana, David Stucki, Christophe Sola, Sebastien Gagneux, Voahangy Rasolofo Razanamparany

**Affiliations:** 1 Mycobacteria Unit, Institut Pasteur of Madagascar, Antananarivo, Madagascar, Ecole Doctorale Science de la Vie et de l’Environnement, Faculté des Sciences, Université d’Antananarivo, Antananarivo, Madagascar; 2 Mycobacteria Unit, Institut Pasteur of Madagascar, Antananarivo, Madagascar; 3 Department of Medical Parasitology and infection Biology, Swiss Tropical and Public Health Institute, Basel, Switzerland; 4 University of Basel, Basel, Switzerland; 5 Institut for Integrative Cell Biology, I2BC, UMR9198 CEA-CNRS-UP Saclay, Orsay, France; Institut de Pharmacologie et de Biologie Structurale, FRANCE

## Abstract

**Background:**

Combining different molecular typing methods for *Mycobacterium tuberculosis* complex (MTBC) can be a powerful tool for molecular epidemiology-based investigation of TB. However, the current standard method that provides high discriminatory power for such a combination, mycobacterial interspersed repetitive units-variable numbers of tandem repeats typing (MIRU-VNTR), is laborious, time-consuming and often too costly for many resource-limited laboratories. We aimed to evaluate a reduced set of loci for MIRU-VNTR typing in combination with spoligotyping and SNP-typing for routine molecular epidemiology of TB.

**Method:**

Spoligotyping and SNP-typing, in combination with the 15 loci MIRU-VNTR typing, were first used to type clinical MTBC isolates (n = 158) from Madagascar. A step by step reduction of MIRU-VNTR loci number was then performed according to the Hunter and Gaston Discriminatory Index (HGDI) and to the Principal component analysis (PCA) correlation with the spoligotype profiles to evaluate the discrimination power inside the generated spoligotype clusters. The 15 MIRU-VNTR was used as reference and SNP-typing was used to determine the main MTBC lineages.

**Results:**

Of the 158 clinical isolates studied, the SNP-typing classified 23 into Lineage 1 (14.6%), 31 into Lineage 2 (19.6%), 23 into Lineage 3 (14.6%) and 81 into Lineage 4 strains (51.3%). 37 different spoligotypes profiles were obtained, 15 of which were unique and 20 in clusters. 15-loci MIRU-VNTR typing revealed 144 different genotypes: 132 isolates had a unique MIRU-VNTR profile and 27 isolates were grouped into 12 clusters. After a stepwise reduction of the MIRU-VNTR loci number within each main spoligotype families, three different sets composed of 5 customised MIRU-VNTR loci had a similar discrimination level to the reference 15 loci MIRU-VNTR in lineage 1, lineage 2 and lineage 3. For lineage 4, a set of 4 and 3 MIRU-VNTR loci were proposed to subtype the Harleem and LAM spoligotype families, respectively. For the T spoligotype family, a set of 5 MIRU-VNTR loci was proposed.

**Conclusion:**

According to the lineages and the spoligotype families, the number of MIRU-VNTR loci can be reduced to get an optimal classification of MTBC. These customized sets of MIRU-VNTR loci reduce workload and save resources while maintaining optimal discriminatory power.

## Introduction

*M*. *tuberculosis* is a widespread global human pathogen whose infection causes an estimated 10.4 million cases of tuberculosis (TB) and 1.8 million deaths every year [1]. TB epidemiology and transmission chain study has traditionally been performed by molecular strain typing [2–4]. Utilizing PCR-based methods for genotyping *M*. *tuberculosis* complex (MTBC) isolates helps public health officials manage TB outbreaks through identification of MTBC population involved in outbreaks and monitoring of their transmission dynamics [5, 6].

SNP-typing, spoligotyping, and MIRU-VNTR typing are three PCR-based typing method used for molecular epidemiology of MTBC strains. SNP-typing, which is based on single nucleotide changes in specific regions of MTBC genomes, is particularly useful for classifying mycobacterial isolates into the main phylogenetic lineages and sub-lineages of the MTBC depending on the number of SNPs assessed [7]. Spoligotyping reveals the absence or presence of unique spacer sequences located between the direct-repeat (DR) sequences of the Clustered Regularly Interspersed Short Palindromic Repeat (CRISPR) region of MTBC genomes [8]. This method is a membrane-based reverse-line blotting procedure, recently adapted to a high-throughput microbead-based format, whose digital results ease the exchange of data between laboratories [9]. However, it is limited by its discrimination power for epidemiology studies and of homoplasies in the DR region that result from convergent evolution [9, 10].

MIRU-VNTR is used to type minisatellite-like loci in the MTBC genome that show polymorphism in the number of tandem repeats. A wide range of MTBC MIRU-VNTR loci that variably discriminate the MTBC populations in the main bacterial lineages have been evaluated [7, 11]. MIRU-VNTR typing has the advantages in the ease of its execution and the generation of results in a digital format [12–14]. MIRU-VNTR has advantages over spoligotyping in its ability to study the diversity and clonal expansion of particular strains at a relatively high resolution, but it can be tedious, labour-intensive, and time consuming due to the large number of loci to screen, although a recently optimized duplex gel-based procedure reduces the workload [15].

Optimized 12, 15, or 24-locus MIRU-VNTR typing systems have been proposed for international standardization [11]. The 15-loci MIRU-VNTR format evaluated for MTBC molecular epidemiological studies showed that 15 and 24 MIRU-VNTR formats provided a similar level of discrimination [16]. However, the initial selection of the loci used for MIRU-VNTR has been reported as biased towards strains of specific lineages and varies in discrimination power within these main MTBC lineages or populations [7, 11]. Customized MIRU-VNTR loci have been proposed to improve the discrimination resolution in specific Ghanaian MTBC populations [14]. Moreover, different studies reported that a combination of MIRU-VNTR typing and spoligotyping offers a better tool for assessment of -TB epidemiology [12, 13, 17]. The combination of both spoligotyping and MIRU-VNTR methods has been reported to provide a similar resolving power relative to the labour-intensive procedure of IS*6110* restriction fragment length polymorphism (RFLP) previously used as a standard for the study of TB transmission [18].

The main goal of this study was to evaluate the combination of spoligotyping with a customized and reduced MIRU-VNTR loci set compared to the reference 15 loci MIRU-VNTR for four out of seven main MTBC lineages (lineage 1, 2, 3 and 4) that are present in Madagascar for an optimal classification of those MTBC strains.

## Materials and methods

### Clinical isolates

Clinical MTBC strains that had previously been typed by spoligotyping were selected in this study to represent the major spoligotype families in Madagascar [8]. Studied strains were selected from the collections of the Mycobacterial unit of the Institute Pasteur of Madagascar. Briefly, 31 strains from Lineage 2 (Beijing), 23 strains from Lineage 3 (Central Asian, CAS), 23 strains from Lineage 1 (East African Indian, EAI), and 81 from Lineage 4 (Euro-American, specifically 18 strains of Haarlem (H), 17 strains of Latino-American (LAM) and 46 strains from the T default spoligotype signature) were studied.

### SNP-typing method

The major phylogenetic lineage of MTBC isolates was determined by SNP-typing using TaqMan simplex real time PCR as previously described [19]. This method is performed to screen SNPs on six genomic regions (*katG*463, Rv2952, Rv3221c, Rv3804c, *gyrA*1842 and *Psts*1) S1 Table. Briefly, 10 ng of DNA was added to a final volume of 12μl containing 5μl TaqMan Universal Master Mix II (Qiagen), 0.05 pmol of wild type probe labelled by FAM and mutant probe labelled by VIC, and 0.83 pmol of each reverse and forward primers for targeted regions S2 Table. The mix reaction was run in a StepOne thermocycler (Applied Biosystems) under the following conditions: 60°C for 30 sec, 95°C 10 minutes, 95°C for 15 sec and 60°C for 1 min for 40 cycles; 60°C for 30 sec. Fluorescence intensities from the VIC and FAM channels were measured at the end of every cycle. Alleles were obtained with the StepOne software (Applied Biosystems)[19].

### MIRU-VNTR typing

MIRU-VNTR typing was initially performed using 15 MIRU-VNTR loci as previously described [11]. Briefly, the 15 MIRU-VNTR loci were amplified in separate PCRs with the primers described in S3 Table. Reaction volumes of 15μl contained 1.5μl of 10X Q solution, 2μl of 5 μM primer set, 2 μl (4mM) of dNTP, 0.1 μl of *Taq* polymerase (0.5 unit) (Eurobio), 0.75μl of DMSO (100%), 3 μl (5M) of betaïne and 3.65 μl of pure H2O. Template DNA (10ng) was added to each PCR mix. *M*. *tuberculosis* H37Rv was included in each set of reactions as a positive control and sterile distilled water as a negative non-template control. Amplification was performed in a thermocycler (Techne, Flexigene) with an initial denaturation step of 94°C for 5 min, followed by 35 cycles of: 94°C for 30 sec, 61°C for 30 sec (55°C for Mtub04, Mtub39, Qub11b, Qub4156), and 70°C for 45 sec. Qub26 had an additional cycle of 70°C for 1 min and 30 sec prior to the cycle of 70°C for 45 sec. The final extension was 72°C for 7 min. The amplified products were visualized by 2% agarose gel electrophoresis. The sizes of the amplicons were estimated by comparison with 100 bp molecular ladders [20]. The allele assignment was determined by comparison with a reference allelic table as given in S4 Table. A cluster of MTBC strains was thus defined as two or more strains with identical MIRU-VNTR genotype patterns.

To search for the concordance between spoligotyping and 15 MIRU-VNTR typing, the 15 MIRU-VNTR profiles obtained from the strains tested were identified in the MIRU-VNTR*plus* database accessible at https://info-demo.lirmm.fr/tbminer/ [21, 22].

### Determination of MTBC spoligotype family and lineage specific MIRU-VNTR loci

Principal component analysis (PCA) based on the 15 MIRU-VNTR data from the studied clinical MTBC strains was performed to determine which group of MIRU-VNTR loci had the ability to identify a specific spoligotype family. The spoligotype family column was used as supplementary variables which had no influence on the principal components of the analysis. The correlation coefficient matrix of the selected MIRU-VNTR loci was generated based on its allele frequency data. The first two principal components (PCs) that explain most of the variation were used to plot the 15 MIRU-VNTR loci in a two-dimensional scatter plot. PCA were performed in package FactoMineR of R software (https://www.r-project.org/).

### HGDI calculation and determination of minimal set of MIRU-VNTR loci

The calculation of the discriminatory power of every studied typing method was based on Simpson’s index of diversity as described by Hunter and Gaston [23]. This value is commonly referred to as the Hunter-Gaston discriminatory index (HGDI) and was calculated by using the Web tool http://insilico.ehu.es/minitools/discriminatory power. HGDI was used to quantify the discriminatory power of each typing method used in isolation or combination and to evaluate the allelic diversity of the different MIRU-VNTR loci. Based on their respective HGDI score, the discriminatory powers were classified as high (HGDI≥0.6), moderate (0.3≤HGDI≤0.6) or low (HGDI≤ 0.3) [20, 24, 25].

To identify a minimal set of MIRU-VNTR loci with an equal discriminatory power as the entire 15 MIRU-VNTR loci set, the loci were ranked in increasing HGDI order and all highly discriminant loci (HGDI ≥ 0.6) were selected; then the other MIRU-VNTR loci were added in a stepwise manner until the HGDI of the reference 15 loci MIRU-VNTR was attained [26].

## Results

### Concordance and discriminatory power of the different genotyping methods

The 158 MTBC strains were divided into four major phylogenetic lineages after genotyping with the 6 SNP regions. In all, SNP-typing assigned 23 isolates into the lineage 1 (14.6%), 31 into lineage 2 (19.6%), 23 into lineage 3 (14.6%) and 81 into lineage 4 (51.3%). Concordance was observed with the classification made with spoligotyping in all strains except for in one which was classified as lineage 3 by spoligotyping but was classified into both lineage 2 and lineage 3 by SNP-typing, possibly suggesting a mixed infection with two strains of MTBC.

When comparing the genotypes obtained with spoligotyping with the 15 MIRU-VNTR typing, concordance was found between spoligotyping and 15 MIRU-VNTR typing for 148 strains (93.7%), while no concordance was found for 10 (6.3%) strains. Full concordance was found for 31 Beijing and 18 Haarlem strains. For CAS, EAI, LAM and T spoligotype family strains, concordances of 95.7%, 87%, 82.4%, 93.5% were observed, respectively. One strain that was classified in both lineage 2 and lineage 3 by the SNP-typing was classified as Beijing by MIRU-VNTR typing. Three strains classified as EAI (13%) and 3 strains classified as LAM (17.6%) by spoligotyping were classified, respectively, as LAM and Haarlem by 15 MIRU-VNTR typing. Three strains classified by spoligotyping as T were classified as Haarlem (n = 2) and EAI (n = 1) by 15 MIRU-VNTR.

Within the 158 strains, 15 MIRU–VNTR typing identified 142 different genotypes. Twenty-seven isolates were grouped into 12 clusters containing 2 to 3 isolates each. Moreover, 132 isolates had a unique MIRU–VNTR profile. Spoligotyping alone identified 35 genotypes, 15 of which were unique, and 20 clusters that contained 2 to 31 strains each. 15 MIRU-VNTR typing combined with either spoligotyping or SNP-typing showed the same discrimination level as 15 MIRU-VNTR typing used alone (HGDI = 0.999).

Thirty-one Beijing strains that clustered by spoligotyping were divided into 29 different MIRU-VNTR genotypes. Of these, 27 MIRU-VNTR genotypes were unique and only 4 strains were grouped into two different clusters. Twenty-three strains belonging to EAI spoligotype family were divided into 22 MIRU-VNTR genotypes, 21 of them were unique and one cluster containing 2 strains. Twenty-three strains of the CAS spoligotype family was divided into 21 different MIRU-VNTR genotypes, 19 of which were unique and 4 formed two clusters each containing 2 strains. Eighteen strains of the Haarlem spoligotype family were distributed into unique MIRU-VNTR genotypes. Seventeen strains with LAM spoligotype profiles were divided into 13 different MIRU-VNTR genotypes, 10 of which were unique and the remaining was grouped into three clusters containing 2 to 3 strains. Forty-six strains with T spoligotype profiles were separated into 34 unique MIRU-VNTR genotypes and four clusters containing 2 to 4 strains (S1 Fig).

### Discriminatory power of the 15 MIRU-VNTR loci to separate the spoligotype families

To define the MIRU-VNTR loci that could be used to classify strains within the spoligotype families, the HGDI of the 15 MIRU-VNTR was calculated for every spoligotype family. According to the global HGDI calculated from the 158 studied strains, 12 MIRU-VNTR loci were designated as “highly discriminant” (HGDI ≥0.6) which were, in order of decreasing discriminatory power, MIRU26, Qub11b, Mtub21, ETR-A, Qub26, Mtub39, MIRU40, MIRU10, Mtub30, Qub4156, ETR-E and MIRU16; while ETR-C, Mtub04 and ETR-D were designated as “moderately discriminant” (0.3≤HGDI≤0.6) (Table 1).

**Table 1:** Global HGDI and HGDI of each locus for spoligotyping family.

LOCI	global HGDI	BEIJING	CAS	EAI	H	LAM	T
MIRU26	0,7893	0,5312	0,1630	0,0833	0,2457	0,5882	0,6690
Qub11b	0,7759	0,6817	0,3007	0,3116	0,7415	0,7132	0,6841
Mtub21	0,7733	0,2430	0,2391	0,3080	0,2157	0,2279	0,4530
ETR-A	0,7629	0,4000	0,1630	0,5942	0,1111	0,5956	0,5528
Qub26	0,7533	0,2452	0,3696	0,7029	0,6928	0,7426	0,5459
Mtub39	0,7362	0,0641	0,4312	0,6268	0,3137	0,6176	0,7340
MIRU 40	0,6982	0,0645	0,4397	0,3007	0,6993	0,7206	0,5424
MIRU10	0,6943	0,1849	0,7754	0,1594	0,7386	0,4853	0,4321
Mtub30	0,6801	0,1269	0,1630	0,2355	0,5425	0,5809	0,2915
Qub4156	0,6455	0,2409	0,5217	0,6051	0,7059	0,4191	0,3240
ETR-E	0,6442	0,2123	0,3007	0,4203	0,1111	0,2206	0,2218
MIRU16	0,6266	0,6172	0,5181	0,1630	0,3660	0,5809	0,7294
ETR-C	0,5744	0,2796	0,4312	0,1630	0,2157	0,1117	0,5575
Mtub04	0,5732	0,5333	0,2391	0,0000	0,4926	0,5809	0,3659
ETR-D	0,372	0,0645	0,1594	0,5471	0,0000	0,1117	0,2449

HGDI ≤ 0.3: low discriminatory power. 0.3 ≤ HGDI ≤ 0.6: moderate discriminatory power. HGDI g 0.6: high discriminatory power.

Within the Beijing family, the loci Qub11b and MIRU16 were highly discriminant for strain classification; the loci Mtub04, MIRU26 and ETR-A were moderately discriminant and the others were poorly discriminant (Table 1). Within the CAS family, only MIRU10 had high discriminatory power, while the loci Qub4156, MIRU16, MIRU40, ETR-C, Mtub39, Qub26 and Qub11b were moderately discriminant and the other loci were poorly discriminant. The loci Qub26, Mtub39 and Qub4156 were highly discriminant for the EAI family while other loci were poorly discriminant. Other highly discriminant loci include Qub11b, MIRU10, Qub4156, MIRU40 and Qub26 for the Haarlem family, Qub26, MIRU40, Qub11b and Mtub39 for the LAM family, and Mtub39, MIRU16, Qub11b and MIRU26 for the T family (Table 1).

### MIRU-VNTR loci and specificities within MTBC spoligotype families

**Fig 1:**
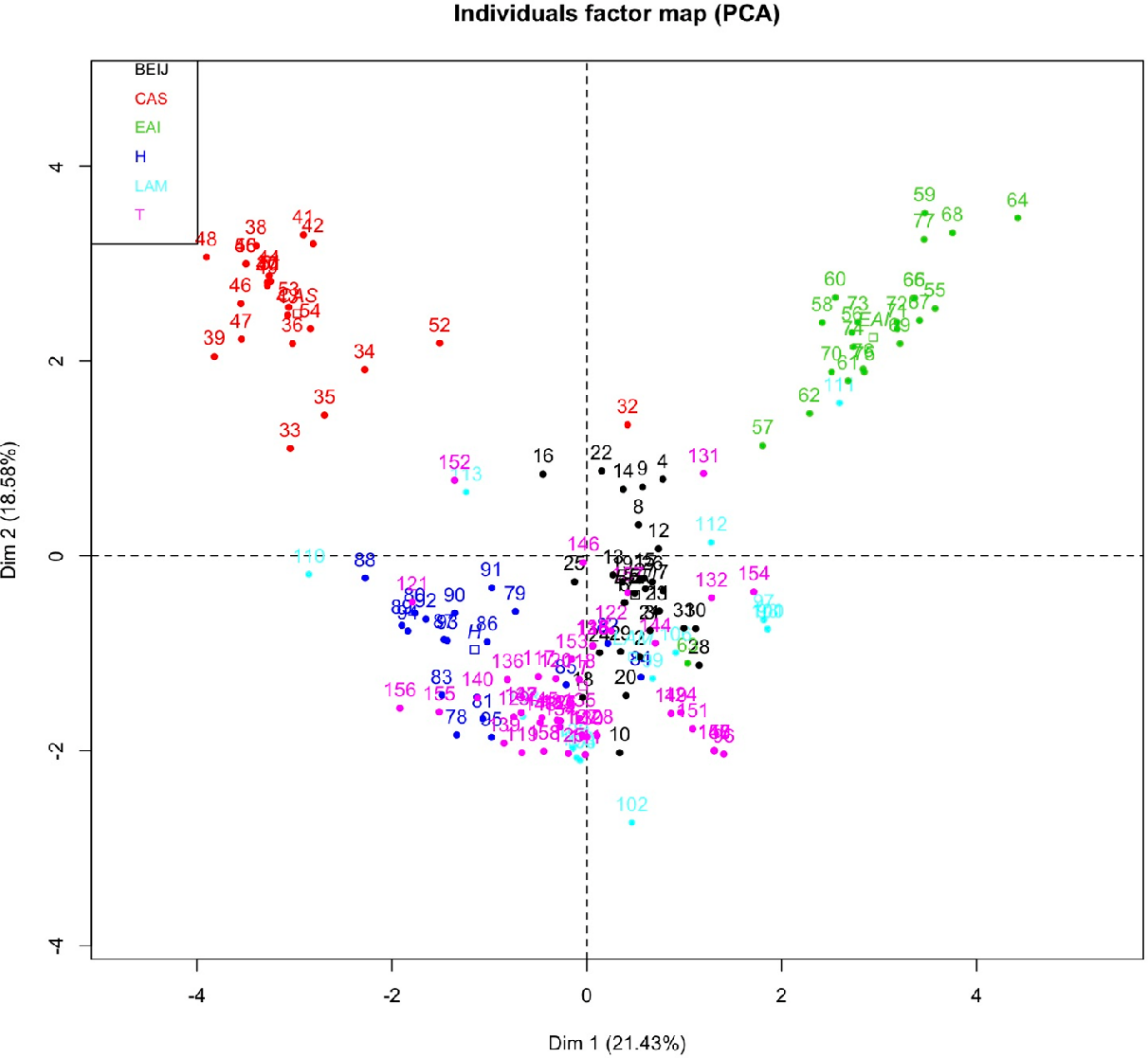
A two dimensional scatter plot based on principal component analysis (PCA) based on 15 MIRU-VNTR individual alleles. MTBC strains having similarity in MIRU-VNTR allelic tend to clusters together.

**Fig 2:**
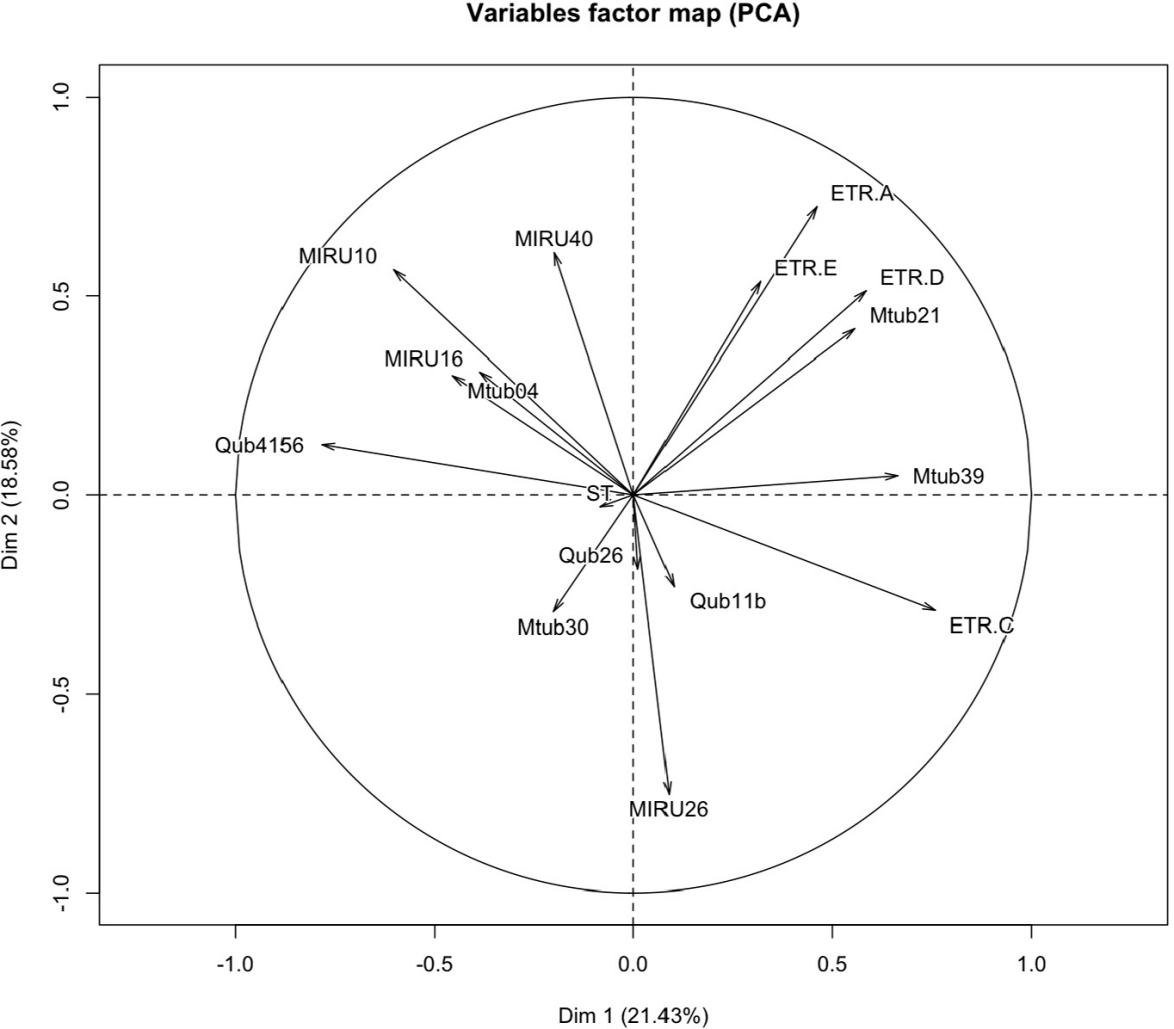
A two dimensional scatter plot based on principal component analysis (PCA) based on 15 MIRU-VNTR individual alleles. MIRU-VNTR marker having similarity in MIRU-VNTR allelic tend to clusters together.

Principal component analysis (PCA) based on 15 MIRU-VNTR data from the studied clinical MTBC strains revealed three major clusters of MTBC strains. The individual plot distinguished the CAS and the EAI spoligotype family that formed two distinct groups while the Beijing, Harleem, LAM and T spoligotype families were grouped into a cloud (Fig 1). According to this PCA, the combination of MIRU10, MIRU16, MIRU40, Mtub04 and Qub4156 and the combination of ETR-A, ETR-D, ETR-E, Mtub21 and Mtub39 were associated to the CAS and EAI spoligotype families, respectively. The combination of ETR-C, MIRU26, Mtub30, Qub11b and Qub26 was associated with the group of Beijing, Harleem, LAM, and T spoligotype families (Fig 2). A set of loci (ETR-C, MIRU26, Mtub30, Qub11b, and Qub26) proposed by PCA can be screened in the first line for Beijing and T families. However, other MIRU-VNTR loci combinations determined by the HGDI method can be added if the HGDI of the reference is not achieved. The combination of MIRU10, MIRU16, MIRU40, Mtub04 and Qub4156, and the combination of ETR-A, ETR-D, ETR-E, Mtub21 and Mtub39 were clustered together and tend to have similar overall alleles for strains of CAS and EAI spoligotype families, respectively. The combination of ETR-C, MIRU26, Mtub30, Qub11b and Qub26 formed a cluster and had similar overall alleles for the group of Beijing, Harleem, LAM, and T spoligotype families. These MIRU-VNTR markers were found in the set of loci proposed based on HGDI and could be done in priory. Per the HGDI value, the combination of Qub26, MIRU40 and Qub11b and the combination of Qub11b, MIRU10, Qub4156, MIRU40 could be suitable alternatives to 15 MIRU-VNTR schemes for subtyping LAM and Harleem spoligotype families, respectively. Based on these results, we have proposed a customized scheme for genotyping MTBC circulating in Madagascar.

### Reduced MIRU-VNTR loci number for subtyping MTBC spoligotype families

To identify a set of loci needed to achieve optimal discrimination, the loci that showed the highest individual discriminatory power were combined with spoligotyping. The MIRU-VNTR loci were added stepwise until the combination of loci showed an HGDI equal to the HGDI of the reference 15 loci MIRU-VNTR.

**Fig 3:**
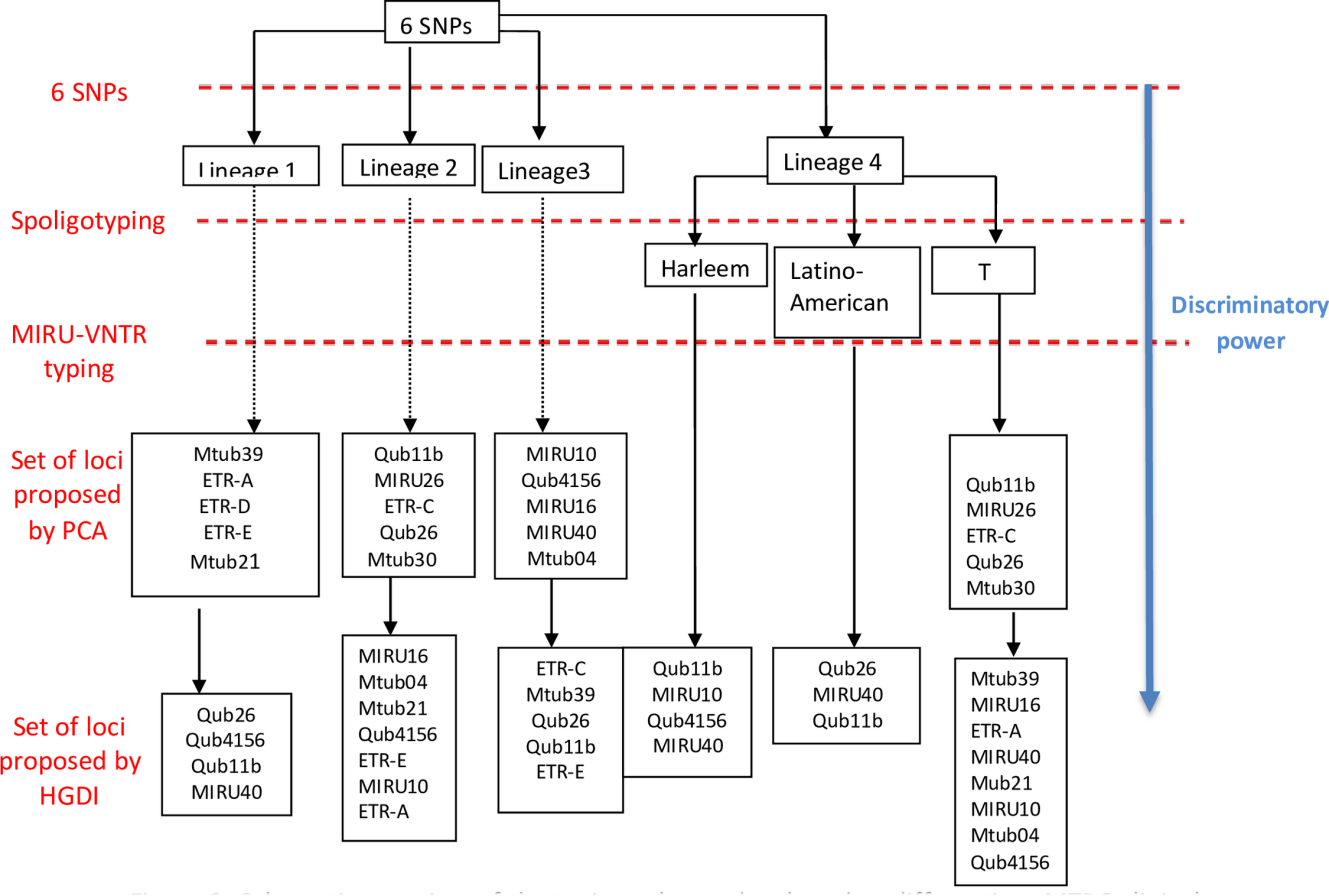
Schematic overview of the typing scheme developed to differentiate MTBC clinical isolates studied. SNPs: Single Nucleotide Polymorphism. The blue arrow indicated that the discrimination level increased with the number of markers typed.

For the Beijing spoligotype family, the reduction to twelve MIRU-VNTR loci (Qub11b, MIRU16, Mtub04, MIRU26, ETR-A, ETR-C, Qub26, Mtub21, Qub4156, ETR E, MIRU10 and Mtub30) showed the same level of discrimination as the 15 loci reference (Table 2). For the EAI spoligotype family, the combination of nine MIRU-VNTR loci (Qub26, Mtub39, Qub4156, ETR-A, ETR-D, ETR-E, Qub11b, Mtub21 and MIRU40) showed the same discrimination level as the 15 loci MIRU-VNTR reference. In the CAS spoligotype family, the combination of ten loci (MIRU10, Qub4156, MIRU16, MIRU40, ETR-C, Mtub39, Qub26, Qub11b, ETR-E and Mtub04) were able to identify single genotype or cluster similar to those obtained with 15 loci reference. For the Haarlem family, four loci (Qub11b, MIRU10, Qub4156 and MIRU40) achieved the same discrimination level as the 15 loci reference (HGDI = 1). For the LAM family, a combination of 3 loci (Qub26, MIRU40 and Qub11b) achieved a similar discrimination level to the 15 loci reference (HGDI = 0.963) (Table 2). Using the above HGDI calculation method, no novel combinations of MIRU-VNTR loci were equivalent in discriminatory power to the 15 MIRU-VNTR for the T family (Fig 3).

**Table 2:** Discrimination power of MIRU-VNTR genetic marker combination proposed.

Spoligotype family	Number of strains tested (n)	MIRU-VNTR loci set (s)	HGDI
Beijing	31	15 MIRU-VNTR	0.996
		**Qub11b, MIRU16, Mtub04,MIRU26, ETR-A, ETR-C,Qub26,Mtub21,Qub4156,ETR E,MIRU10,Mtub30**	**0.996**
EAI	23	15 MIRU-VNTR	0.996
		**Qub26, Mtub39,Qub4156,ETR-A, ETR-D, ETR-E,Qub11b,Mtub21,MIRU40**	**0.996**
CAS	23	15 MIRU-VNTR	0.991
		**MIRU10,Qub4156, MIRU16,MIRU40, ETR-C, Mtub39,Qub26,Qub11b,ETR-E,Mtub04**	**0.991**
Haarlem	19	15 MIRU-VNTR	1
		**Qub11b, MIRU10, Qub4156,MIRU40**	**1**
LAM	17	15 MIRU-VNTR	0,963
		**Qub26, MIRU40, Qub11b**	**0.963**
T	45	15 MIRU-VNTR	0.988
		Mtub39, MIRU16,Qub11b, MIRU26, ETR-C, ETR-A Qub26, MIRU40, Mtub21, MIRU10, Mtub04, Qub4156 and Mtub30	0.976

In bold, the set of MIRU-VNTR markers combination proposed by HGDI method for each spoligotype family.

## Discussion

The main goal of this study was to evaluate the combination of spoligotyping with a reduced number of MIRU-VNTR loci that would have an equivalent discriminatory power as the 15 MIRU-VNTR typing method. This combination is expected to improve on the use of MIRU-VNTR alone in which the selected loci were reported to be biased towards lineage 4 and can lack discriminatory power for other lineages [7]. This is the first study to use such a combination to describe the genetic diversity of Malagasy MTBC clinical isolates.

To confirm the MTBC lineages classification obtained by spoligotyping, the related profiles were compared to those obtained by the more robust SNP-typing to make sure that the spoligotyping profiles were not prone to homoplasy. Our results showed 99% concordance between spoligotyping and SNP-typing for the 158 MTBC strains studied. The spoligotyping could be used in the first line to identify the main sublineages of the studied strains. Then, for the lineages 1, 2 and 3, sets of MIRU-VNTR loci proposed by this study could be directly screened. However, for the lineage 4, which grouped three spoligotype families, the spoligotyping must be done before screening the set of MIRU-VNTR. This may improve the discrimination power of the combined methods against the initial biased selection of the used MIRU-VNTR loci toward this MTBC lineage 4. Spoligotyping can be more laborious when added to the customized set of 3 to 11 MIRU-VNTR lineage-depending loci proposed. However, the 15-MIRU-VNTR method alone needs from 9 to 15 PCR runs without the automatized equipment in low-resource laboratory, as some loci can be screened in duplex PCR. Moreover, it has been suggested by this study and others that combining both methods can be much more discriminant in clusterizing clinical MTBC strains [12, 13]. Spoligotyping and MIRU-VNTR typing have their own database and identifying strains genotype using the combination with customized loci proposed here can be performed using softwares like the MIRUVNTR-plus used in present study where customized MIRU, with or without combination with Spoligotyping can be used to subtype the strains.

The unique spoligotype profile that was discordant with SNP-typing could be indicative of a mixed infection; spoligotyping classified the strain as CAS (lineage 3) while SNP-typing classified it into lineage 2 and 3. Additional genotyping of this particular strain with MIRU-VNTR typing gave a unique Beijing profile that suggested probable homoplasy generated by the spoligotyping, possibly due to convergent evolution. Similar discordances between spoligotyping and alternative genotyping methods was described by Fenner et al., particulary in the case of “Pseudo-Beijing” strains [27].

As previously reported in the literature, a higher discrimination power was found for the 15 MIRU-VNTR typing compared to the spoligotyping alone [12, 13]. A 6.3% discordance was found between the spoligotyping and MIRU-VNTR typing alone; this observation is in agreement with other studies showing differences between spoligotyping and MIRU-VNTR typing in classifying MTBC strain. This discordance could possibly be explained by the clinical isolates representing mixed infections, that is, to say, an infection with two or more different genotypes. Multiple infections implying high reinfection rates and the absence of efficient immunity conferred to the host by the initial infection were indeed frequently reported [28]. This discordance might also be explained by convergent evolution of the spoligotypes, however, the SNP-typing obtained lineages did not differ from the spoligotyping ones and this SNP-spoligotyping had been selected to classify the strains due to the stability of the SNPs [18].

This is the first study to investigate a minimal set of MIRU-VNTR loci for Malagasy spoligotype families. Similar studies trying to define a minimal set of loci to classify MTBC strains suggested a fixed set of MIRU-VNTR loci (MIRU10, MIRU16, Mtub04, Mtub21, Mtub39, ETR-B, Qub11b and Qub26) in combination with spoligotyping for epidemiological study [29]. While these loci were found to be highly discriminate in our Malagasy strains, combinations of varied MIRU-VNTR loci individualized to each spoligotype family and based on HGDI were found to be more discriminative than a fixed set of MIRU-VNTR loci. Another study conducted to genotype MTBC population in Ghana proposed the customized Ghanaian lineage-specific set of MIRU10, MIRU40, ETR-A, Qub11b, Qub26 and Qub4156 to subtyping the lineage 4 and 5 MTBC from this country [14]. The loci described from the Ghanaian study were comprised of sets of loci for subtyping the Malagasy spoligotypes families. This study is also consistent with a PHRANA (progressively hierarchical resolving analysis using nucleic acid) scheme as described by P. Keim on the anthrax model [30]. The proposed combination of both methods can thus be used to improve the discriminatory power to classify MTBC populations in a wider range of MTBC population in different countries. Customized specific reduced number of MIRU-VNTR loci could be proposed to subtype spoligotype families without a loss in discrimination compared to the 15 loci MIRU-VNTR. The spoligotype specific MIRU-VNTR loci identified by PCA were found in the set of loci proposed based on HGDI method. A set of MIRU-VNTR loci identified by PCA can be typed and added to the other more discriminant loci proposed by the HGDI method.

The present study revealed that, due to the strong phylogeographic structure exhibited by MTBC, the most relevant MIRU-VNTR typing schemes will likely differ depending on the specific geographical setting. For example, in Northwest Russia, MIRU26 and ETR-A were proposed for the Beijing family while, in South Africa, MIRU10, MIRU26 and MIRU39 were proposed for the same family [31].

Due to the fact that the original selections of the 24 loci and the derived 15 loci widely used for MIRU-VNTR were biaised toward some main lineages, the MIRU-VNTR technique may be also biased depending on the most predominant strains in the country where the method is performed. Combining the spoligotyping and MIRU-VNTR typing can avoid such a bias as this combination can be performed independently from countries or regions from where the strains were isolated.

The studied strains were representing only the major spoligotype families circulating in Madagascar. The proposed customized combination has to be evaluated in multicentric studies in settings with different MTBC strain distributions to validate its resolution and ability to discriminate MTBC in different settings. Indeed, to assess the whole typeability and variability of loci, more samples by spoligotype family should have been taken in the present study. Twenty strains by major spoligotype family were randomly selected here without taking account Share-Type (ST); then the ability of these combination of loci proposed to distinguish strains of the same ST was not evaluated. Moreover, this study was limited to 15 MIRU-VNTR; while evaluating the 24 loci would have increased the chance of having more hypervariability. This customized combination of genotyping methods should be evaluated as a first-line panel for genotyping MTBC isolates in a particular setting. It is more cost effective than the full set of 15 loci for molecular epidemiological investigation of TB transmission in those resource-limited settings.

## Conclusion

The use of combined spoligotyping and customized MIRU-VNTR loci proposed in this study to subtype MTBC isolated in Madagascar offered a similar discriminatory power as the 15-loci MIRU-VNTR typing used alone. The optimal combination of genetic markers proposed in this study achieved an acceptable level of resolution for classifying MTBC strains during tuberculosis transmission surveys. This test proves to be less laborious and resource demanding than MIRU-VNTR loci alone and would be particularly useful in low-resource settings.

## Supporting information

S1 Fig Concordance between spoligotyping and 15 MIRU-VNTR typing. Beijing, CAS, EAI, H, LAM, T: spoligotype family were identified by spoligotyping; A: Identification of strain, B: MtbC15-9 type assigned by MIRU-VNTR*plus*, C: from left to right, ETR-D, MIRU10, MIRU16, MIRU26, ETR-E,MIRU40, Mtub04, Mtub21, Mtub30, Mtu39,ETR-A, ETR-C, Qub11b, Qub26 and Qub4156.
(TIF)

S1 Table SNPs (n = 6) or mutations characteristic of *M*. *tuberculosis* lineage. AA: Amono-Acide, SNP: Single Nucleotide Polymorphism, H37Rv: reference strain of *M*. *tuberculosis*, In bold the nucleotide muted.
(PDF)

S2 Table Primer and probe sequences for TaqMan SNPs typing assays. (PDF)

S3 Table 15 loci MIRU-VNTR combination as described by Supply et al, 2006 [10]. ^a^ Position of the locus in H37rv genome, ^b^ Name of the locus.
(PDF)

S4 Table Table showing the amplicon size and corresponding number of units (allele) in the questioned locus (Christophe Sola, 2009; interne communication). In bold the expected size for H37rv.
(PDF)

## References

[1] WHO. Global tuberculosis report 2015 WHO Report 2015. 2015(WHO/HTM/TB/2015).

[2] Thumamo BP, Asuquo AE, Abia-Bassey LN, Lawson L, Hill V, Zozio T, et al Molecular epidemiology and genetic diversity of *Mycobacterium tuberculosis* complex in the Cross River State, Nigeria. Infect Genet Evol. 2011 6;12(4):671–7. doi: 10.1016/j.meegid.2011.08.011 21878397 10.1016/j.meegid.2011.08.011 PMC3369698

[3] Rasolofo-Razanamparany V, Menard D, Ratsitorahina M, Auregan G, Gicquel B, Chanteau S. Transmission of tuberculosis in the prison of Antananarivo (Madagascar). Res Microbiol. 2000 11;151(9):785–95. 11130869 10.1016/s0923-2508(00)01144-x

[4] Mathema B, Kurepina NE, Bifani PJ, Kreiswirth BN. Molecular epidemiology of tuberculosis: current insights. Clin Microbiol Rev. 2006 10;19(4):658–85. doi: 10.1128/CMR.00061-05 17041139 10.1128/CMR.00061-05 PMC1592690

[5] Moss AR, Hahn JA, Tulsky JP, Daley CL, Small PM, Hopewell PC. Tuberculosis in the homeless. A prospective study. Am J Respir Crit Care Med. 2000 8;162(2 Pt 1):460–4. 10934071 10.1164/ajrccm.162.2.9910055

[6] Saavedra-Campos M, Welfare W, Cleary P, Sails A, Burkitt A, Hungerford D, et al Identifying areas and risk groups with localised *Mycobacterium tuberculosis* transmission in northern England from 2010 to 2012: spatiotemporal analysis incorporating highly discriminatory genotyping data. Thorax. 2010 4 28. 10.1136/thoraxjnl-2014-206416 25920328

[7] Comas I, Homolka S, Niemann S, Gagneux S. Genotyping of genetically monomorphic bacteria: DNA sequencing in *Mycobacterium tuberculosis* highlights the limitations of current methodologies. PLoS One. 2009;4(11):e7815 doi: 10.1371/journal.pone.0007815 19915672 10.1371/journal.pone.0007815 PMC2772813

[8] Kamerbeek J, Schouls L, Kolk A, van Agterveld M, van Soolingen D, Kuijper S, et al Simultaneous detection and strain differentiation of *Mycobacterium tuberculosis* for diagnosis and epidemiology. J Clin Microbiol. 1997 4;35(4):907–14. 9157152 10.1128/jcm.35.4.907-914.1997 PMC229700

[9] Sola C. Clustured regularly interspersed short palindromic repeats (CRISPR) genetic diversity studies as a mean to reconstruct the evolution of the *Mycobacterium tuberculosis* complex. Tuberculosis (Edinb). 2015 6;95 Suppl 1:S159–66. 25748060 10.1016/j.tube.2015.02.029

[10] Stucki D, Ballif M, Egger M, Furrer H, Altpeter E, Battegay M, et al Standard Genotyping Overestimates Transmission of *Mycobacterium tuberculosis* among Immigrants in a Low-Incidence Country. J Clin Microbiol. 2016 7;54(7):1862–70. doi: 10.1128/JCM.00126-16 27194683 10.1128/JCM.00126-16 PMC4922098

[11] Supply P, Allix C, Lesjean S, Cardoso-Oelemann M, Rusch-Gerdes S, Willery E, et al Proposal for standardization of optimized mycobacterial interspersed repetitive unit-variable-number tandem repeat typing of *Mycobacterium tuberculosis*. J Clin Microbiol. 2006 12;44(12):4498–510. doi: 10.1128/JCM.01392-06 17005759 10.1128/JCM.01392-06 PMC1698431

[12] Streit E, Baboolal S, Akpaka PE, Millet J, Rastogi N. Finer characterization of *Mycobacterium tuberculosis* using spoligotyping and 15-loci MIRU-VNTRs reveals phylogeographical specificities of isolates circulating in Guyana and Suriname. Infect Genet Evol. 2014 3;30:114–9. doi: 10.1016/j.meegid.2014.12.015 25528138 10.1016/j.meegid.2014.12.015

[13] Chaoui I, Zozio T, Lahlou O, Sabouni R, Abid M, El Aouad R, et al Contribution of spoligotyping and MIRU-VNTRs to characterize prevalent *Mycobacterium tuberculosis* genotypes infecting tuberculosis patients in Morocco. Infect Genet Evol. 2014 1;21:463–71. doi: 10.1016/j.meegid.2013.05.023 23732366 10.1016/j.meegid.2013.05.023

[14] Asante-Poku A, Nyaho MS, Borrell S, Comas I, Gagneux S, Yeboah-Manu D. Evaluation of customised lineage-specific sets of MIRU-VNTR loci for genotyping *Mycobacterium tuberculosis* complex isolates in Ghana. PLoS One. 2014;9(3):e92675 doi: 10.1371/journal.pone.0092675 24667333 10.1371/journal.pone.0092675 PMC3965448

[15] Vasconcellos SE, Acosta CC, Gomes LL, Conceicao EC, Lima KV, de Araujo MI, et al Strain classification of *Mycobacterium tuberculosis* isolates in Brazil based on genotypes obtained by spoligotyping, mycobacterial interspersed repetitive unit typing and the presence of large sequence and single nucleotide polymorphism. PLoS One. 2014;9(10):e107747 doi: 10.1371/journal.pone.0107747 25314118 10.1371/journal.pone.0107747 PMC4196770

[16] Silva C, Perdigao J, Jordao L, Portugal I. Mycobacterial interspersed repetitive unit typing and mutational profile for multidrug-resistant and extensively drug-resistant tuberculosis surveillance in Portugal: a 3-year period overview. Int J Antimicrob Agents. 2014 12;44(6):546–51. doi: 10.1016/j.ijantimicag.2014.06.021 25270633 10.1016/j.ijantimicag.2014.06.021

[17] Lu W, Lu B, Liu Q, Dong H, Shao Y, Jiang Y, et al Genotypes of *Mycobacterium tuberculosis* isolates in rural China: using MIRU-VNTR and spoligotyping methods. Scand J Infect Dis. 2014 2;46(2):98–106. doi: 10.3109/00365548.2013.858182 24359517 10.3109/00365548.2013.858182

[18] Maes M, Kremer K, van Soolingen D, Takiff H, de Waard JH. 24-locus MIRU-VNTR genotyping is a useful tool to study the molecular epidemiology of tuberculosis among Warao Amerindians in Venezuela. Tuberculosis (Edinb). 2008 9;88(5):490–4. 18514577 10.1016/j.tube.2008.04.003

[19] Stucki D, Malla B, Hostettler S, Huna T, Feldmann J, Yeboah-Manu D, et al Two new rapid SNP-typing methods for classifying *Mycobacterium tuberculosis* complex into the main phylogenetic lineages. PLoS One. 2012;7(7):e41253 doi: 10.1371/journal.pone.0041253 22911768 10.1371/journal.pone.0041253 PMC3401130

[20] Supply P, Mazars E, Lesjean S, Vincent V, Gicquel B, Locht C. Variable human minisatellite-like regions in the *Mycobacterium tuberculosis* genome. Mol Microbiol. 2000 5;36(3):762–71. 10844663 10.1046/j.1365-2958.2000.01905.x

[21] Allix-Beguec C, Harmsen D, Weniger T, Supply P, Niemann S. Evaluation and strategy for use of MIRU-VNTRplus, a multifunctional database for online analysis of genotyping data and phylogenetic identification of *Mycobacterium tuberculosis* complex isolates. J Clin Microbiol. 2008 8;46(8):2692–9. doi: 10.1128/JCM.00540-08 18550737 10.1128/JCM.00540-08 PMC2519508

[22] Aze J, Sola C, Zhang J, Lafosse-Marin F, Yasmin M, Siddiqui R, et al Genomics and Machine Learning for Taxonomy Consensus: The *Mycobacterium tuberculosis* Complex Paradigm. PLoS One. 2015;10(7):e0130912 doi: 10.1371/journal.pone.0130912 26154264 10.1371/journal.pone.0130912 PMC4496040

[23] Hunter PR, Gaston MA. Numerical index of the discriminatory ability of typing systems: an application of Simpson's index of diversity. J Clin Microbiol. 1988 11;26(11):2465–6. 3069867 10.1128/jcm.26.11.2465-2466.1988 PMC266921

[24] Sola C, Filliol I, Legrand E, Lesjean S, Locht C, Supply P, et al Genotyping of the *Mycobacterium tuberculosis* complex using MIRUs: association with VNTR and spoligotyping for molecular epidemiology and evolutionary genetics. Infect Genet Evol. 2003 7;3(2):125–33. 12809807 10.1016/s1567-1348(03)00011-x

[25] Cowan LS, Diem L, Monson T, Wand P, Temporado D, Oemig TV, et al Evaluation of a two-step approach for large-scale, prospective genotyping of *Mycobacterium tuberculosis* isolates in the United States. J Clin Microbiol. 2005 2;43(2):688–95. doi: 10.1128/JCM.43.2.688-695.2005 15695665 10.1128/JCM.43.2.688-695.2005 PMC548083

[26] Luo T, Yang C, Pang Y, Zhao Y, Mei J, Gao Q. Development of a hierarchical variable-number tandem repeat typing scheme for *Mycobacterium tuberculosis* in China. PLoS One. 2014;9(2):e89726 doi: 10.1371/journal.pone.0089726 24586989 10.1371/journal.pone.0089726 PMC3934936

[27] Fenner L, Malla B, Ninet B, Dubuis O, Stucki D, Borrell S, et al "Pseudo-Beijing": evidence for convergent evolution in the direct repeat region of *Mycobacterium tuberculosis*. PLoS One. 2011;6(9):e24737 doi: 10.1371/journal.pone.0024737 21935448 10.1371/journal.pone.0024737 PMC3172296

[28] Warren RM, Victor TC, Streicher EM, Richardson M, Beyers N, Gey van Pittius NC, et al Patients with active tuberculosis often have different strains in the same sputum specimen. Am J Respir Crit Care Med. 2004 3 01;169(5):610–4. doi: 10.1164/rccm.200305-714OC 14701710 10.1164/rccm.200305-714OC

[29] Zhang J, Heng S, Le Moullec S, Refregier G, Gicquel B, Sola C, et al A first assessment of the genetic diversity of *Mycobacterium tuberculosis* complex in Cambodia. BMC Infect Dis. 2011;11:42 doi: 10.1186/1471-2334-11-42 21299851 10.1186/1471-2334-11-42 PMC3062598

[30] Keim P, Van Ert MN, Pearson T, Vogler AJ, Huynh LY, Wagner DM. Anthrax molecular epidemiology and forensics: using the appropriate marker for different evolutionary scales. Infect Genet Evol. 2004 9;4(3):205–13. doi: 10.1016/j.meegid.2004.02.005 15450200 10.1016/j.meegid.2004.02.005

[31] Mokrousov I, Narvskaya O, Limeschenko E, Vyazovaya A, Otten T, Vyshnevskiy B. Analysis of the allelic diversity of the mycobacterial interspersed repetitive units in *Mycobacterium tuberculosis* strains of the Beijing family: practical implications and evolutionary considerations. J Clin Microbiol. 2004 6;42(6):2438–44. doi: 10.1128/JCM.42.6.2438-2444.2004 15184416 10.1128/JCM.42.6.2438-2444.2004 PMC427846

