# Supplementary figures and images for: Evaluation of spoligotyping, SNPs and customised MIRU-VNTR combination for genotyping Mycobacterium tuberculosis clinical isolates in Madagascar
Source: PLoS One. 2017 Oct 20;12(10):e0186088. doi: 10.1371/journal.pone.0186088 (PMC5650158; doi:10.1371/journal.pone.0186088)

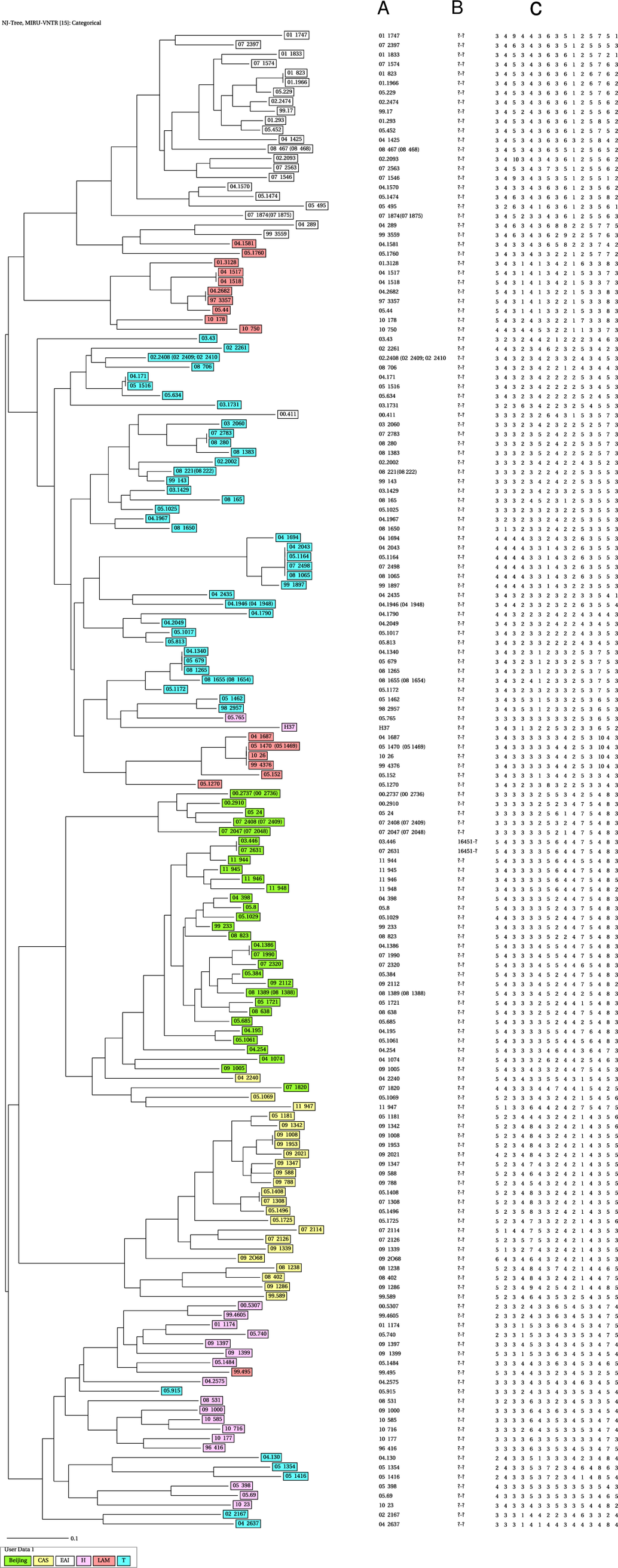

Supplement: S1 Fig — Beijing, CAS, EAI, H, LAM, T: spoligotype family were identified by spoligotyping; A: Identification of strain, B: MtbC15-9 type assigned by MIRU-VNTRplus, C: from left to right, ETR-D, MIRU10, MIRU16, MIRU26, ETR-E,MIRU40, Mtub04, Mtub21, Mtub30, Mtu39,ETR-A, ETR-C, Qub11b, Qub26 and Qub4156. (TIF) [file pone.0186088.s001.tif]
