# Supplementary material for: Evaluation of spoligotyping, SNPs and customised MIRU-VNTR combination for genotyping Mycobacterium tuberculosis clinical isolates in Madagascar
Source: PLoS One. 2017 Oct 20;12(10):e0186088. doi: 10.1371/journal.pone.0186088 (PMC5650158; doi:10.1371/journal.pone.0186088)
Supplement: S1 Table — AA: Amono-Acide, SNP: Single Nucleotide Polymorphism, H37Rv: reference strain of M. tuberculosis, In bold the nucleotide muted. (PDF) [file pone.0186088.s002.pdf]

Lineage	Name of SNPs	Name of gene	Nucleotide position	codon	Nucleotide in H37Rv	AA in H37Rv	Nucleotide in Mutant	AA in Mutant
Euro-American (Lineage4)	*katG*463	*katG* or Rv1908c	1389	463	C**G**G	Arg	C**T**G	Leu
East-Asian (Lineage2)	Rv2952_0526n	Rv2952	526	176	**G**GG	Gly	**A**GG	Arg
Indo-oceanic (Lineage1)	Rv3221c_0085n	TB.3 or Rv3221c	85	29	**G**TC	Val	**A**TC	Ile
East-African-Indian (Lineage3)	Rv3804c_0012n	*fbpA* or Rv3804c	12	4	GT**T**	Val	GT**C**	Val
TbD1	*gyrA*1842	*gyrA* or Rv0006	1842	614	AT**T**	Ile	AT**C**	Ile
Bovis-Mada	*PstS*1_1055	*PstS*1 or Rv0934	1055	352	G**T**T	Val	G**C**T	Ala

S1 Table. SNPs (n=6) or mutations characteristic of *M. tuberculosis* lineage. AA: Amono-Acide, SNP: Single Nucleotide Polymorphism, H37Rv: reference strain of *M. tuberculosis,* In bold the nucleotide muted
