# Supplementary material for: Evaluation of spoligotyping, SNPs and customised MIRU-VNTR combination for genotyping Mycobacterium tuberculosis clinical isolates in Madagascar
Source: PLoS One. 2017 Oct 20;12(10):e0186088. doi: 10.1371/journal.pone.0186088 (PMC5650158; doi:10.1371/journal.pone.0186088)
Supplement: S2 Table — (PDF) [file pone.0186088.s003.pdf]

Lineage	Name of SNPs	Forward primer_sequence	Reveres_primer _sequence	Wild type allele probe	Mutant allele probe
Euro-American	*katG*463	CCGAGATTGCCAGCCTTAAG	GAAACTAGCTGTGAGACAGTCAATCC	6FAM-CCAGATCCTGGCATC	VIC-CAGATCCGGGCATC
East-Asian	Rv2952_0526n	CCTTCGATGTTGTGCTCAATGT	CATGCGGCGATCTCATTGT	6FAM-CCCAGGAGGGTAC	VIC-CCCAGGAAGGTACT
Indo-oceanic	Rv3221c_0085n	TGTCAACGAAGGCGATCAGA	GACCGTTCCGGCAGCTT	6FAM-ACAAGGGCGACGTC	VIC-ACAAGGGCGACATC
East-African-Indian	Rv3804c_0012	GCATGGATGCGTTGAGATGA	CGAGTCGACGCGACATACC	6FAM-AAGAATGCAGCTTGTTGA	VIC-AAGAATGCAGCTTGTCGA
TBD1 MADA	gyrA1842	CCAGCCCGAGGAACGCATCGCCCA	AGCACCAGGTACGGGGCGTCGG	6FAM-TCCAGATTCGCGGCT	VIC-TCCAGATCCGCGGCT
Bovis_Mada	Psts1_1055	CACCGACGGCAACAAGGCCTC	TTCACCACCGCGGGCGGCAGCGGCT	6-FAM-ACCAGGTTCATTTCC	VIC-ACCAGGCTCATTTCC

S2 Table. Primer and probe sequences for TaqMan SNPs typing assays
