# Supplementary material for: Evaluation of spoligotyping, SNPs and customised MIRU-VNTR combination for genotyping Mycobacterium tuberculosis clinical isolates in Madagascar
Source: PLoS One. 2017 Oct 20;12(10):e0186088. doi: 10.1371/journal.pone.0186088 (PMC5650158; doi:10.1371/journal.pone.0186088)
Supplement: S3 Table — a Position of the locus in H37rv genome, b Name of the locus. (PDF) [file pone.0186088.s004.pdf]

MIRU - VNTR locus [(a)]	MIRU - VNTR alias [(b)]	Forward primer 5' to 3'	Reverse primer 5' to 3'
580	MIRU04 (ETR–D)	ATTTCGATCGGGATGTTGAT	TCGGTCCCATCACCTTCTTA
960	MIRU10	GTTCTTGACCAACTGCAGTCGTCC	GCCACCTTGGTGATCAGCTACCT
1644	MIRU16	TCGGTGATCGGGTCCAGTCCAAGTA	CCCGTCGTGCAGCCCTGGTAC
2996	MIRU26	CCCGCCTTCGAAACGTCGCT	TGGACATAGGCGACCAGGCGAATA
3192	MIRU31 (ETR–E)	ACTGATTGGCTTCATACGGCTTTA	GTGCCGACGTGTCTTGAT
802	MIRU40	GGGTTGCTGGATGACAACGTGT	GGGTGATCTCGGCGAAATCAGATA
424	Mtub04	GTCCAGGTTGCAAGAGATGG	GGCATCCTCAACAACGGTAG
1955	Mtub21	AGATCCCAGTTGTCGTCGTC	CAACATCGCCTGGTTCTGTA
2401	Mtub30	AGTCACCTTTCCTACCACTCGTAAC	ATTAGTAGGGCACTAGCACCTCAAG
3690	Mtub39	AATCACGGTAACTTGGGTTGTTT	GATGCATGTTCGACCCGTAG
2165	ETR–A	ATTTCGATCGGGATGTTGAT	TCGGTCCCATCACCTTCTTA
577	ETR–C	GACTTCAATGCGTTGTTGGA	GTCTTGACCTCCACGAGTGC
2163b	Qub-11b	CGTAAGGGGGATGCGGGAAATAGG	CGAAGTGAATGGTGGTGGCAT
4052	Qub-26	GGCCAGGTCCTTCCCGAT	AACGCTCAGCTGTCGGAT
4156	Qub4156	TGACCACGGATTGCTCTAGT	GCCGGCGTCCATGTT

S3 Table. 15 loci MIRU-VNTR combination as described by Supply et al, 2006 [10]. [a] Position of the locus in H37rv genome, [b] Name of the locus
