# Supplementary material for: Evaluation of spoligotyping, SNPs and customised MIRU-VNTR combination for genotyping Mycobacterium tuberculosis clinical isolates in Madagascar
Source: PLoS One. 2017 Oct 20;12(10):e0186088. doi: 10.1371/journal.pone.0186088 (PMC5650158; doi:10.1371/journal.pone.0186088)
Supplement: S4 Table — In bold the expected size for H37rv. (PDF) [file pone.0186088.s005.pdf]

MIRU - VNTR alias	ETR A	ETR B	ETR C	ETRD/MIRU04	ETR E/MIRU31	MIRU 02	MIRU10	MIRU 16	MIRU 20	MIRU 23	MIRU 24	MIRU 26	MIRU 27	MIRU 39	MIRU 40	Mtub04	Mtub 21	Mtub 29	Mtub 30	Mtub 34	Mtub 39	Qub11b	Qub26	Qub4156
MIRU - VNTR locus (unit size in pb)	2165 75pb)	2461 (57pb)	0577 (58pb)	0580 (77pb)	3192 (53pb)	0154 (53pb)	0959 (53pb)	1644 (53pb)	2059 (77pb)	2531 (53pb)	2687 (53pb)	2996 (51pb)	3007 (53pb)	4348 (53pb)	0802 (54pb)	0424 (51pb)	1955 (57pb)	2347 (57pb)	2401 (58pb)	3171 (54pb)	3690 (58pb)	2163 (69pb)	4052 (111pb)	4156 (59pb)
Allele																								
1	247	178	172	176	545	455	535	618	514	607	**447**	511	551	593	**407**	177	149	179	261	171	225	136	264	563
2	322	235	230	253	598	**508**	590	**671**	**591**	661	500	562	604	**646**	461	218	**206**	238	**319**	225	283	205	375	622
3	**397**	**292**	288	**330**	**651**	561	**643**	724	668	714	553	**613**	**657**	699	515	**269**	263	293	377	**279**	341	274	486	**681**
4	472	349	**346**	407	704	614	696	777	745	767	606	664	709	752	569	320	320	350	435	333	399	343	597	740
5	547	406	404	484	757	667	749	829	822	820	659	715	762	805	623	371	377	407	493	387	457	**412**	**708**	799
6	622	463	462	561	810	720	802	882	873	**873**	712	766	815	911	677	422	434	464	551	441	**515**	481	819	858
7	697	520	520	638	863	773	855	935	976	926	765	817	868	964	731	473	491	521	609	495	573	550	930	917
8	772	577	578	715	916	826	908	988		979	818	868	921		785	524	548	578	667	549	631	619	1041	
9	847	634	636	792		879	961	1041			871	919			839	575	605	635	725	603	747	688		
10	922	691	684	869		932	1013				924				893	626	662	692	783	657		757		

S4 Table. Table showing the amplicon size and corresponding number of units (allele) in the questioned locus (Christophe Sola, 2009; interne communication). In bold the expected size for H37rv
